# Comparative Precision of 3D MRE and 2D MRE for Measurement of Liver Stiffness in Adults with Severe Obesity

**DOI:** 10.1148/radiol.253243

**Published:** 2026-05-05

**Authors:** Lukas Müller, Lael Ceriani, David T. Harris, Tanya Wolfson, Danielle Batakis, Rashmi Agni, Yesenia Covarrubias, Kay Pepin, Richard L. Ehman, Jeremiah Heilman, Nikolaos Panagiotopoulos, Gavin Hamilton, Michael S. Middleton, Vitor F. Martins, Anthony C. Gamst, Ryan Sappenfield, Eduardo Grunvald, Luke M. Funk, Garth R. Jacobsen, Anne O. Lidor, James A. Goodman, Sami B. Khoury, Claude B. Sirlin, Scott B. Reeder

**Affiliations:** ^1^Department of Radiology, University of Wisconsin-Madison, 600 Highland Ave, Madison, WI 53792; ^2^Department of Diagnostic and Interventional Radiology, University Medical Center Mainz, Mainz, Germany; ^3^Department of Radiology, University of California San Diego, San Diego, Calif; ^4^Computational and Applied Statistical Laboratory (CASL), San Diego Supercomputer Center, University of California San Diego, San Diego, Calif; ^5^Department of Pathology and Laboratory Medicine, University of Wisconsin-Madison, Madison, Wis; ^6^Resoundant, Rochester, Minn; ^7^Department of Radiology, Mayo Clinic, Rochester, Minn; ^8^Department of Mathematics, University of California San Diego, San Diego, Calif; ^9^Department of Medicine, University of California San Diego, San Diego, Calif; ^10^Department of Surgery, University of Wisconsin-Madison, Madison, Wis; ^11^Department of Surgery. William S. Middleton VA, Madison, Wis; ^12^Department of Surgery, University of California San Diego, San Diego, Calif; ^13^Translational Clinical Sciences, Pfizer Research & Development, Cambridge, Mass; ^14^Department of Medical Physics, University of Wisconsin-Madison, Madison, Wis; ^15^Department of Biomedical Engineering, University of Wisconsin-Madison, Madison, Wis; ^16^Department of Medicine, University of Wisconsin-Madison, Madison, Wis; ^17^Department of Emergency Medicine, University of Wisconsin-Madison, Madison, Wis

## Abstract

**Background:**

The comparative precision of two-dimensional (2D) MR elastography (MRE) and three-dimensional (3D) MRE for measuring liver stiffness (LS) in adults with severe obesity is unknown.

**Purpose:**

To compare test-retest repeatability, between-day reproducibility, and between–field-strength reproducibility of 2D MRE– and 3D MRE–based LS measurements in adults with severe obesity.

**Materials and Methods:**

In this prospective dual-center study (December 2020 to August 2023), adults with severe obesity underwent 2D MRE and 3D MRE at one visit before weight loss surgery and two visits after. At each visit, MRE was repeated with interexamination repositioning at 3 T to assess test-retest repeatability, or at 1.5 T and 3 T to assess between–field-strength reproducibility. Some participants also underwent MRE 1–3 days before visit 1 to assess between-day reproducibility at 1.5 T or 3 T. All participant visits were performed at the same site. Absolute repeatability coefficient (RC) and proportional RC (RC%), absolute reproducibility coefficient (RDC) and proportional RDC (RDC%), intraclass correlation coefficient (ICC), and technical failure rates were computed for 2D MRE–based and 3D MRE–based LS and compared pairwise (bootstrap-based tests or McNemar test of paired proportions for clustered data, as appropriate).

**Results:**

In a total of 103 participants (mean age, 44 years ± 10.0; 89 women), LS ranges were 2.2 kPa ± 0.4 with 2D MRE and 2.0 kPa ± 0.3 with 3D MRE. At 3 T, 3D MRE had better test-retest repeatability than did 2D MRE (RC, 0.26 vs 0.55 kPa, respectively; RC%, 12.6% vs 19.9%; ICC, 0.85 vs 0.67; all *P* < .001). Three-dimensional MRE had better between–field-strength reproducibility than did 2D MRE (RDC, 0.26 vs 0.38 kPa, respectively; RDC%, 14.6% vs 18.5%; both *P* < .001). Three-dimensional MRE had better between-day reproducibility than did 2D MRE at 3 T (RDC, 0.22 vs 0.48 kPa, respectively; *P* = .007), but not at 1.5 T. However, 3D MRE had a higher failure rate than did 2D MRE (4.9% [29 of 587] vs 2.9% [17 of 583]; *P* = .007).

**Conclusion:**

Compared with 2D MRE, 3D MRE had better precision for measuring liver stiffness in adults with severe obesity but failed more frequently.

ClinicalTrials.gov identifier: NCT03674528

© The Author(s) 2026. Published by the Radiological Society of North America under a CC BY 4.0 license.

*Supplemental material is available for this article.*

See also the editorial by Hu in this issue.

## Graphical abstract

**Figure:**
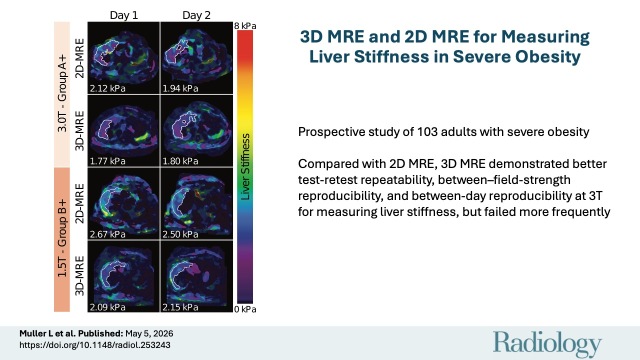

**Summary**

Compared with two-dimensional MR elastography (MRE), three-dimensional MRE demonstrated better test-retest repeatability, between–field-strength reproducibility, and between-day reproducibility at 3 T for measurement of liver stiffness in adults with severe obesity but failed more frequently.

**Key Results**

■ In a prospective study of 103 adults with severe obesity, three-dimensional (3D) MR elastography (MRE) had better test-retest repeatability for measuring liver stiffness at 3 T than did two-dimensional (2D) MRE (repeatability coefficient, 0.26 vs 0.55 kPa; proportional repeatability coefficient, 12.6% vs 19.9%; intraclass correlation coefficient, 0.85 vs 0.67; all *P* < .001).
■ Three-dimensional MRE had better between–field-strength reproducibility than did 2D MRE (reproducibility coefficient [RDC], 0.26 vs 0.38 kPa, respectively; *P* < .001) and between-day reproducibility at 3 T (RDC, 0.22 vs 0.48 kPa; *P* = .007), but not at 1.5 T (RDC, 0.26 vs 0.30 kPa; *P* = .53).
■ Three-dimensional MRE failed more frequently than did 2D MRE (4.9% [29 of 587] vs 2.9% [17 of 583]; *P* = .007).

## Introduction

Metabolic dysfunction–associated steatotic liver disease (MASLD) is the most common cause of chronic liver disease worldwide (1). MASLD can progress to its more advanced form, metabolic dysfunction–associated steatohepatitis (MASH), which can include fibrosis (2). Patients with precirrhotic fibrotic MASH are at increased risk of developing cirrhosis and other adverse liver outcomes, such as hepatocellular carcinoma and hepatic decompensation (2). The U.S. Food and Drug Administration recently approved two drugs to treat adults with precirrhotic fibrotic MASH: resmetirom and semaglutide (3,4). Other drugs targeting precirrhotic fibrotic MASH are under development (5). Identifying patients with MASH or fibrotic MASH is therefore important.

MR elastography (MRE) is an established, noninvasive imaging method that quantifies liver stiffness (LS) as a biomarker of liver fibrosis. MRE-based LS has higher accuracy for classifying liver fibrosis stage than do transient elastography and clinical prediction rules (6–8). Although MRE is accurate in patients with obesity (9–12) and has high test-retest repeatability and between–field-strength reproducibility in patients without severe obesity (13–18), there is a paucity of data on the precision of MRE in patients with severe obesity. This gap in knowledge is important because obesity is frequently associated with MASLD and fibrotic MASH, degrades the performance of imaging-based tests by reducing signal-to-noise ratio and causing artifacts, and thereby introduces measurement variability. Such variability is clinically important because the difference in LS between different fibrosis stages is small. For example, a meta-analysis by Liang et al (19) suggested LS cut-offs for 2.65 kPa for stage F1 or greater, 3.14 kPa for F2 or greater, and 3.53 kPa for F3 or greater. Hence, even minor variability in LS measurements may shift patients into different fibrosis categories.

Furthermore, data are limited regarding the comparative performance of two-dimensional (2D) MRE and three-dimensional (3D) MRE in adults with severe obesity. Although both 2D MRE and 3D MRE use section-selective 2D encoding to generate raw images, 2D MRE applies motion encoding in a single direction to capture wave motion in individual 2D sections (hence the term *2D*). To compare, 3D MRE captures the wavefield throughout a 3D volume (hence the term *3D*) by applying motion encoding sequentially in each of three orthogonal directions. This allows extraction of viscoelastic biomarkers beyond LS (20) and may improve precision for measuring LS.

We hypothesized that 3D MRE would provide more precise and reproducible LS measurements than would 2D MRE in adults with severe obesity. Therefore, the aim of this prospective dual-center study was to compare test–retest repeatability, between-day reproducibility, and between–field-strength reproducibility of 2D MRE–based and 3D MRE–based LS measurements in adults with severe obesity.

## Materials and Methods

### Study Design and Participants

This prospective study formed part of the second cycle of a larger Health Insurance Portability and Accountability Act–compliant, prospective, dual-center study (site 1: University of California, San Diego; site 2: University of Wisconsin-Madison) for validation of MR biomarkers of obesity-associated MASLD (ClinicalTrials.gov identifier: NCT03674528). The local institutional review boards approved the study, and all participants gave written informed consent. A recent report included diagnostic accuracy data from this study (21). Overlap includes participants and research data, but none of the repeatability and reproducibility results of the current work were reported. This study received funding from Pfizer to support staff and investigator effort, study procedures, and participant stipends, and in-kind support from Resoundant, which provided centralized automated analysis of MRE data, blinded to other study data. GE HealthCare provided general research support to both sites, not specific to the current study.

**Figure 1:**
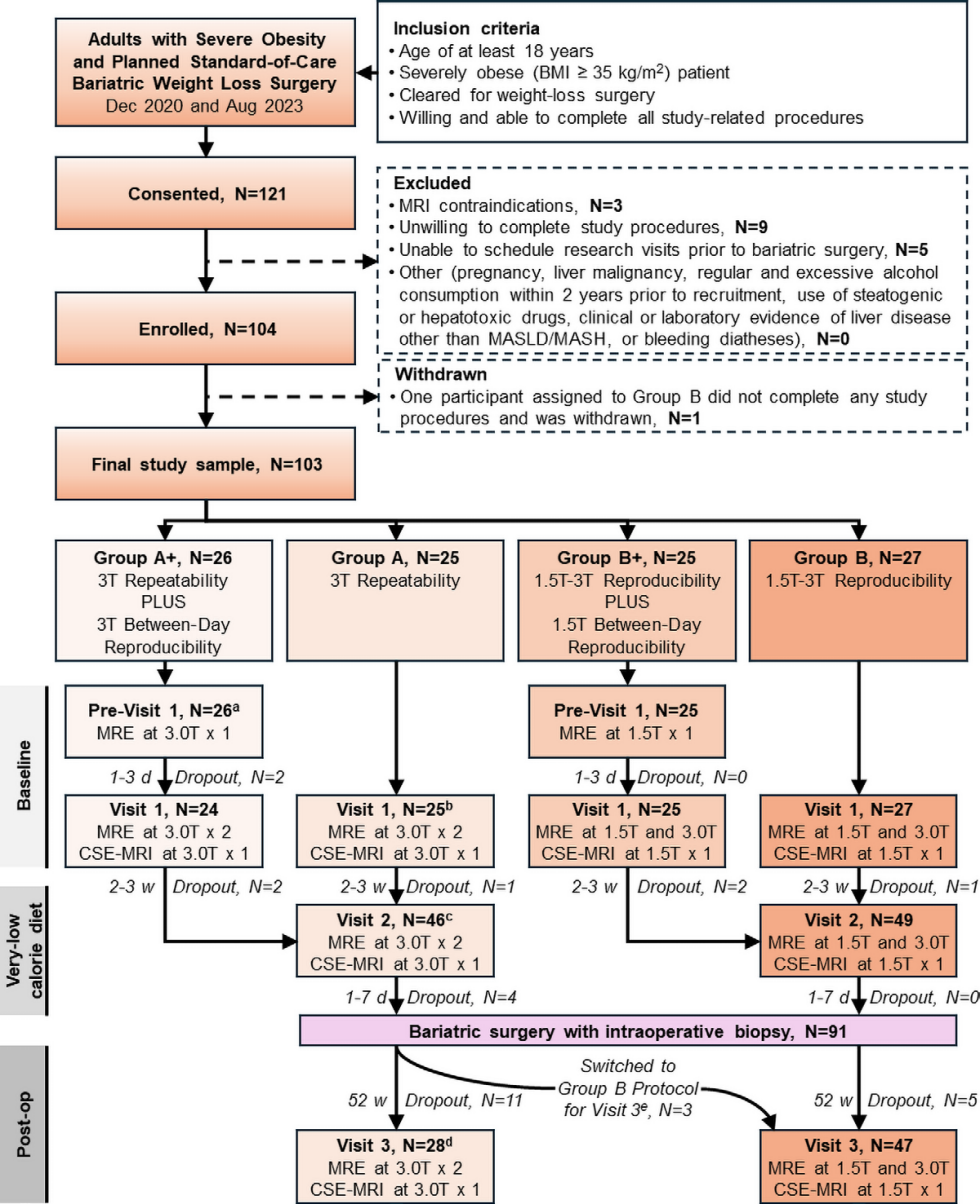
Consolidated Standards of Reporting Trials (known as CONSORT) flow diagram shows the study design, including withdrawals after consent and after enrollment. ^a^ Two participants had no repeated two-dimensional (2D) and three-dimensional (3D) MR elastography (MRE) (MRI system failure, *n* = 1; claustrophobia reaction, *n* = 1). ^b^ Two participants had no repeated 2D and 3D MRE (coordinator error, *n* = 1; scheduling constraint, *n* = 1); one participant had no repeated 3D MRE (participant discomfort, *n* = 1;). ^c^ One participant had no repeated 2D and 3D MRE (co-ordinator error, *n* = 1). ^d^ One participant had no repeated 3D MRE (MR system went down, *n* = 1). ^e^ Three participants in group A were switched to the group B protocol for visit 5 (coordinator error, *n* = 3). CSE = chemical shift encoded, MASH = metabolic dysfunction–associated steatohepatitis, MASLD = metabolic dysfunction–associated steatotic liver disease.

The Consolidated Standards of Reporting Trials (known as CONSORT) flow diagram of participant selection is illustrated in Figure 1. Between December 2020 and August 2023, adults aged 18 years or older with severe obesity (body mass index ≥ 35 [calculated as weight in kilograms divided by height in meters squared]) and with planned standard-of-care bariatric surgery (sleeve gastrectomy or gastric bypass) were recruited (target enrollment ≥100). Further inclusion criteria were ability and willingness to complete all study-related procedures. Exclusion criteria were contraindications to MRI, unwillingness to complete study procedures, pregnancy or planned pregnancy during the study period, known liver malignancy, regular and excessive alcohol consumption within 2 years before recruitment, use of steatogenic or hepatotoxic medications, clinical or laboratory evidence of liver disease other than MASLD or nonalcoholic steatohepatitis, and bleeding diathesis. The target sample size was based on accuracy-related aims of the study, which required a minimum of 85 participants at visit 2. On the basis of anticipated dropout and failure rates, a target sample size of 100 or greater was proposed to achieve that requirement.

Participants were divided into four groups. Site 1 (which had 3-T systems only) enrolled group A and A+ participants. Participants were initially assigned consecutively to group A+ until enrollment targets were met and subsequently to group A. Site 2 (which had both 1.5-T and 3-T systems) enrolled group B and B+ participants. Participants were initially assigned consecutively to group B+ until enrollment targets were met and subsequently to group B. Enrollment at site 1 lagged, so site 2 enrolled participants into group A after completing group B enrollment.

Each participant underwent three research visits. Visit 1 occurred 2–4 weeks before surgery, before initiation of the presurgery restricted-calorie liquid diet (22). Visit 2 occurred after the liquid diet and 1–7 days before surgery. Visit 3 occurred 12 months after surgery. At each visit, MRE was repeated at the same field strength (3 T) with interexamination repositioning to assess test-retest repeatability (groups A and A+) or at different field strengths (1.5 T, 3 T) to assess between–field-strength reproducibility (groups B and B+). Group A+ and B+ participants also underwent previsit 1, performed 1–3 days before visit 1, with MRE performed at 3 T (group A+) or 1.5 T (group B+). Data from previsit 1 were paired with data from visit 1 to assess between-day reproducibility at 3 T (group A+) or 1.5 T (group B+). Chemical shift–encoded MRI was performed at all visits to estimate proton density fat fraction and R2*. A research wedge biopsy specimen was collected during surgery. For each participant, all visits and study-related procedures were conducted at the same site.

### MRI and MRE Procedures

Imaging was conducted by using clinical MRI systems at 1.5 T (Signa Artist; GE HealthCare [at site 2]) and/or 3 T (Signa Premier [at site 2] or Signa Discovery 750 [at site 1]; GE HealthCare). At each site, each 1.5-T or 3-T examination was performed with the same system. Participants fasted for at least 4 hours. At site 1, a 32-element torso phased-array coil was used before March 15, 2023, and Adaptive Image Receive (AIR; GE HealthCare) coils thereafter. At site 2, AIR coils were used throughout. Each MRI examination was performed by one of several study-trained technologists, who followed standardized imaging protocols and completed predefined procedural checklists.

Both 2D MRE and 3D MRE were performed using a flexible passive driver placed over the right liver lobe and secured using an elastic band with vibrations at 60 Hz. Use of 2D MRE was implemented with an acquisition sequence equivalent to the commercial version of 2D MRE on the GE platform. Use of 3D MRE was implemented with a research implementation developed at the Mayo Clinic (23,24). Acquisition parameters are listed in Table S1. At 2D MRE, elastography data were acquired at four section locations in the liver. At 3D MRE, data were acquired at 32 section locations, but the analysis was confined to four central sections. Participants were removed from the table and repositioned, and localizer acquisitions repeated, for test–retest acquisitions.

Complex-based 3D chemical shift–encoded MRI (IDEAL IQ; GE HealthCare) was performed to measure proton density fat fraction and R2* across the liver (21,22) to characterize the cohort.

### Image Analysis

The 2D MRE images were reconstructed offline using a 2D multimodal direct inversion algorithm (25) to generate LS maps. The 3D MRE images were reconstructed offline using a 3D direct inversion algorithm (26) to generate LS, storage modulus (a marker of fibrosis), and loss modulus (a putative marker of inflammation) maps (27). MRE data were analyzed with automated software (Hepatogram+; Resoundant) (24,26) operated by a scientist at Resoundant (K.P., with >10 years of experience in MRE data analysis), blinded to other study data. For 3D MRE, the four central sections were analyzed. The automated method placed a region of interest within the liver on each of the four acquired 2D MRE sections and on each of the four analyzed 3D MRE sections. If the total number of analyzable pixels within the four regions of interest was 500 pixels or greater, the acquisition was considered valid, and the numeric values for LS (2D MRE, 3D MRE) and for the storage and loss moduli (3D MRE) were recorded (23,24). The damping ratio (a putative marker of inflammation) was calculated by dividing the loss modulus by twice the storage modulus (23,24). If the number of analyzable pixels was fewer than 500, the acquisition was considered a failure and the numeric values were discarded, as recommended in the Quantitative Imaging Biomarkers Alliance guidelines (28). Examples of technical failures are provided in Figures S1 and S2.

Proton density fat fraction and R2* analyses were performed by an experienced imaging analyst (D.B., with 8 years of experience in MRI analysis) blinded to other study data.

Information regarding bariatric surgery, histopathologic assessment, and clinical and laboratory evaluations is provided in Appendix S1.

### Statistical Analysis

Statistical analyses were performed by an author (T.W., with >25 years of experience) using statistical software (R, version 2023; The R Foundation for Statistical Computing). Participant characteristics and MRE technical failure rates were summarized descriptively. Technical failure rates pooled over all visits and repeats for 2D MRE and 3D MRE were compared (McNemar test of paired proportions for clustered data). Test–retest repeatability, between-day reproducibility, and between–field-strength reproducibility were assessed for 2D MRE and 3D MRE parameters for cases with relevant pairs of valid MRE acquisitions. Reproducibility between 2D MRE and 3D MRE for measuring LS was assessed as a secondary analysis.

For test-retest repeatability, between–field-strength reproducibility, and between-method data were pooled across visits. Bland-Altman plots were generated, and Quantitative Imaging Biomarkers Alliance (known as QIBA)–recommended precision parameters were computed: bias and its significance; 95% limits of agreement; intraclass correlation coefficient (ICC); absolute repeatability coefficient (RC) or reproducibility coefficient (RDC), defined as 2.77 × within-case coefficient of variability; and proportional RC (RC%) or proportional RDC (RDC%). RC% and RDC% are a constant multiplied by the within-case coefficient of variation, which is a root mean square transformation of the ratio of individual variances to individual means. Although a standardized percentage measure, it does not have a readily interpretable numerator and denominator.

The 95% CIs around ICCs were computed using nonparametric bootstrap to adjust for within-case dependence. The 2D MRE and 3D MRE precision parameters were compared (bootstrap-based tests), applying piece-wise Bonferroni correction to adjust for multiple testing (*P* ≤ .0167 was considered to indicate significance at a family-wise .05 level). Unadjusted *P* ≤ .05 was considered to indicate a statistically significant difference.

## Results

### Participants

In total, 121 potential participants were recruited. Seventeen were excluded due to the following: MRI contraindications (*n* = 3), unwillingness to complete study procedures (*n* = 9), or inability to schedule research visits (*n* = 5) (Fig 1). One participant assigned to group B did not come to their scheduled baseline visit and was withdrawn (*n* = 1). The final study sample consisted of 103 participants (mean age, 44 years ± 10 [SD]; age range, 24–66 years; 89 women): 26 participants in group A+, 25 participants in group A, 25 participants in group B+, and 28 participants in group B. Table 1 summarizes baseline characteristics overall and for each group. Participant race and ethnicity were self-reported using predefined categories in the medical history questionnaire; for the overall cohort, reported ethnicity/race categories were Asian (1%; one of 97), Black or African American (4%; four of 97), Native Hawaiian or other Pacific Islander (1%; one of 97), White (89%; 86 of 97), and more than one race category (5%; five of 97). Ethnicity categories were Hispanic (15.0%; 15 of 100) and non-Hispanic (85.0%; 85 of 100).

**Table 1:** Baseline Characteristics of Participants

Characteristic	All Participants (*n* = 103)	Group A+ (*n* = 26)	Group A (*n* = 25)	Group B+ (*n* = 25)	Group B (*n* = 27)
Demographic					
Age (range) (y)	44 ± 10.0 (24–66)	44 ± 8.9 (27–66)	40 ± 11.2 (25–62)	48 ± 9.7 (30–66)	43 ± 8.9 (24–62)
Sex*					
Female	86.4 (89/103)	76.0 (19/25)	92.0 (23/25)	88.0 (22/25)	89.3 (25/28)
Male	13.6 (14/103)	24.0 (6/25)	8.0 (2/25)	12.0 (3/25)	10.7 (3/28)
Race*					
Asian	1.0 (1/97)	0 (0/22)	4.5 (1/22)	0 (0/25)	0 (0/28)
Black/African American	4.1 (4/97)	4.5 (1/22)	4.5 (1/22)	0 (0/25)	7.1 (2/28)
Native Hawaiian/ other Pacific Islander	1.0 (1/97)	4.5 (1/22)	0 (0/22)	0 (0/25)	0 (0/28)
White	88.7 (86/97)	86.4 (19/22)	72.7 (16/22)	100 (25/25)	92.9 (26/28)
>1 category	5.2 (5/97)	4.5 (1/22)	18.2 (4/25)	0 (0/25)	0 (0/28)
Ethnicity*					
Hispanic	15.0 (15/100)	24.0 (6/25)	37.5 (9/24)	0 (0/24)	0 (0/27)
Non-Hispanic	85.0 (85/100)	76.0 (19/25)	62.5 (15/24)	100 (24/24)	100 (27/27)
Anthropometrics					
BMI†	45.9 ± 7.3	45.9 ± 8.3	41.6 ± 7.1	48.3 ± 6.3	47,7 ± 6.0
Hip circumference (cm)	141.0 ± 14.2	137.6 ± 13.7	133.8 ± 14.4	147.1 ± 11.9	144.7 ± 13.2
Waist circumference (cm)	124.0 ± 14.8	124.3 ± 15.4	118.9 ± 10.1	126.5 ± 14.3	126.2 ± 17.4
Waist-to-hip ratio	0.9 ± 0.1	0.9 ± 0.1	0.9 ± 0.1	0.9 ± 0.1	0.9 ± 0.1
Laboratory values^‡^					
AST (U/L)	21.4 ± 8.4	24.2 ± 11.1	24.2 ± 9.6	18.8 ± 3.6	18.6 ± 5.7
ALT (U/L)	25.6 ± 12.0	27.8 ± 13.7	29.6 ± 15.4	23.5 ± 8.7	22.0 ± 7.9
Triglycerides (mg/dL)	119.1 ± 47.2	118.3 ± 42.7	106.1 ± 38.6	129.1 ± 45.7	122.5 ± 57.9
HDL cholesterol (mg/dL)	44.0 ± 10.7	41.1 ± 10.5	43.3 ± 9.4	43.2 ± 12.5	48.0 ± 9.6
LDL cholesterol (mg/dL)	110.4 ± 30.0	111.2 ± 34.0	105.9 ± 31.1	110.9 ± 33.9	113.4 ± 21.5
Hemoglobin A_1c_ (%)	5.9 ± 1.1	5.8 ± 0.9	5.9 ± 1.5	6.0 ± 0.9	5.8 ± 0.8
Platelet count (×10^3^/μL)	280.8 ± 72.0	282.8 ± 65.1	277.8 ± 86.6	266.8 ± 52.5	295.0 ± 79.3
Imaging results					
PDFF (%)	9.5 ± 7.3	10.9 ± 8.1	9.2 ± 7.2	10.0 ± 7.4	8.1 ± 6.8
R2* (1/sec)	45.5 ± 10.5	45.0 ± 12.1	41.8 ± 10.0	51.0 ± 11.3	44.3 ± 6.9
2D MRE LS (kPa)	2.2 ± 0.4	2.3 ± 0.4	2.2 ± 0.2	2.1 ± 0.3	2.2 ± 0.4
3D MRE LS (kPa)	2.0 ± 0.3	2.1 ± 0.4	2.0 ± 0.2	1.9 ± 0.2	2.0 ± 0.5
Histologic results^§^					
Steatosis grade					
0 (< 5%)	45.1 (41/91)	55.0 (11/20)	36.4 (8/22)	39.1 (9/23)	50.0 (13/26)
1 (5–33%)	47.3 (43/91)	35.0 (7/20)	54.5 (12/22)	52.2 (12/23)	46.2 (12/26)
2 (34%–66%)	5.5 (5/91)	10.0 (2/20)	4.5 (1/22)	4.3 (1/23)	3.8 (1/26)
3 (> 66%)	2.2 (2/91)	0 (0/20)	4.5 (1/22)	4.3 (1/23)	0 (0/26)
Hepatocellular ballooning					
None	75.8 (69/91)	85.0 (17/20)	63.6 (14/22)	73.9 (17/23)	80.8 (21/26)
Mild	24.2 (22/91)	15.0 (3/20)	36.4 (8/22)	26.1 (6/23)	19.2 (5/26)
Lobular inflammation					
0	78.0 (71/91)	75.0 (15/20)	86.4 (19/22)	73.9 (17/23)	76.9 (20/26)
<2 foci per ×20 magnification	19.8 (18/91)	25.0 (5/20)	13.6 (3/22)	21.7 (5/23)	19.2 (5/26)
2–4 foci per ×20 magnification	2.2 (2/91)	0 (0/20)	0 (0/22)	4.3 (1/23)	3.8 (1/26)
Steatohepatitis					
Not MASLD	45.1 (41/91)	55.0 (11/20)	36.4 (8/22)	39.1 (9/23)	50.0 (13/26)
MASLD but not MASH	34.1 (31/91)	30.0 (6/20)	40.9 (9/22)	34.8 (8/23)	30.8 (8/26)
Definite MASH	20.9 (19/91)	15.0 (3/20)	22.7 (5/22)	26.1 (6/23)	19.2 (5/26)
Fibrosis stage					
0	82.4 (75/91)	85.0 (17/20)	81.8 (18/22)	87.0 (20/23)	76.9 (20/26)
1	11.0 (10/91)	5.0 (1/20)	13.6 (3/22)	8.7 (2/23)	15.4 (4/26)
2	5.5 (5/91)	10.0 (2/20)	4.5 (1/22)	4.3 (1/23)	3.8 (1/26)
3	1.1 (1/91)	0 (0/20)	0 (0/22)	0 (0/23)	3.8 (1/26)

Note.—Unless otherwise indicated, data are percentages and data in parentheses are numerators/denominators; mean data are ± SD. Group A and A+: 3.0-T MR elastography (MRE) for assessment of test-retest repeatability with (A+) or without (A) an additional previsit examination for between-day reproducibility. Group B and B+: 1.5-T and 3.0-T MRE for assessment of between–field-strength reproducibility, with (B+) or without (B) an additional previsit examination for between-day reproducibility. Body mass index (BMI) was calculated as weight in kilograms divided by height in meters squared. 2D = two-dimensional, 3D = three-dimensional, ALT = alanine aminotransferase, AST = aspartate aminotransferase, BMI = body mass index, HDL= high-density lipoprotein, LDL = low-density lipoprotein, LS = liver stiffness, MASH = metabolic dysfunction–associated steatohepatitis, MASLD = metabolic dysfunction–associated steatotic liver disease, PDFF = proton density fat fraction.

* The total number of participants varies across rows because one participant assigned to group B did not complete any study procedures and was withdrawn (*n* = 1) and because completion of the medical history questionnaire, including self-reported demographic information, was voluntary.

^‡^ To convert laboratory values to SI units, multiply the values by the following conversion factors: ALT and AST, 0.0167 (to μkat/L); for triglycerides, 0.0113 (to mmol/L); for LDL cholesterol and HDL cholesterol, 0.0259 (to mmol/L); for platelet counts, 1 (to ×10^9^/L).

^§^ Histologic results were available in 91 patients. Lobular inflammation was analyzed at 20× magnification.

Participants underwent a total of 377 visits, with dropouts between visits shown in Figure 1. Three group A participants underwent 1.5-T and 3-T MRE (the protocol intended for group B) at visit 3 because of co-ordinator error. Mean weight and body mass index at each visit are summarized in Table S2.

### MRE Acquisitions and Technical Failure Rates

The 377 visits were intended to have a total of 597 2D MRE and 3D MRE examinations. Of these, 14 2D MRE and 10 3D MRE acquisitions were not attempted (reasons listed in Fig 1). When attempted, 3D MRE had a higher failure rate (4.9%; 29 of 587) overall than 2D MRE (2.9%; 17 of 583) (*P* = .007). Table 2 summarizes failure rates for each method.

**Table 2:** Summary of Technical Success and Failure Rates of 2D and 3D MRE and 3D MRE for Participants across Four Visits

Field Strength by Visit and MRE Method	No. of Attempted Examinations	No. of Failures	No. of Successes
1.5 T			
Previsit 1			
2D	25	0 (0)	25 (100)
3D	25	0 (0)	25 (100)
Visit 1			
2D	53	0 (0)	53 (100)
3D	53	1 (1.9)	52 (98)
Visit 2			
2D	48	0 (0)	48 (100)
3D	48	0 (0)	48 (100)
Visit 3			
2D	47	0 (0)	47 (100)
3D	47	0 (0)	47 (100)
All visits pooled			
2D	173	0 (0)	173 (100.0)
3D	173	1 (0.6)	172 (99.4)
3 T			
Previsit 1			
2D	23	3 (13)	20 (87)
3D	24	3 (12)	21 (87)
Visit 1			
2D	146	8 (5.5)	138 (94.5)
3D	147	14 (9.5)	133 (90.5)
Visit 2			
2D	141	4 (2.8)	137 (97.2)
3D	141	8 (5.7)	133 (94.3)
Visit 3			
2D	100	2 (2.0)	98 (98.0)
3D	102	3 (2.9)	99 (97.1)
All visits pooled			
2D	410	17 (4.1)	393 (95.9)
3D	414	28 (6.8)	386 (93.2)
Pooling both field strengths			
Previsit 1			
2D	48	3 (6)	45 (93)
3D	49	3 (6)	46 (94)
Visit 1			
2D	199	8 (4.0)	191 (96.0)
3D	200	15 (7.5)	185 (92.5)
Visit 2			
2D	189	4 (2.1)	185 (97.9)
3D	189	8 (4.2)	181 (95.8)
Visit 3			
2D	147	2 (1.4)	145 (98.6)
3D	149	3 (2.0)	146 (98.0)
All visits pooled			
2D	583	17 (2.9)	566 (97.1)
3D	587	29 (4.9)	558 (95.1)

Note.—Data in parentheses are percentages. There was a higher overall failure rate for three-dimensional (3D) MR elastography (MRE) compared with two-dimensional (2D) MRE.

### Repeatability Analysis

**Figure 2:**
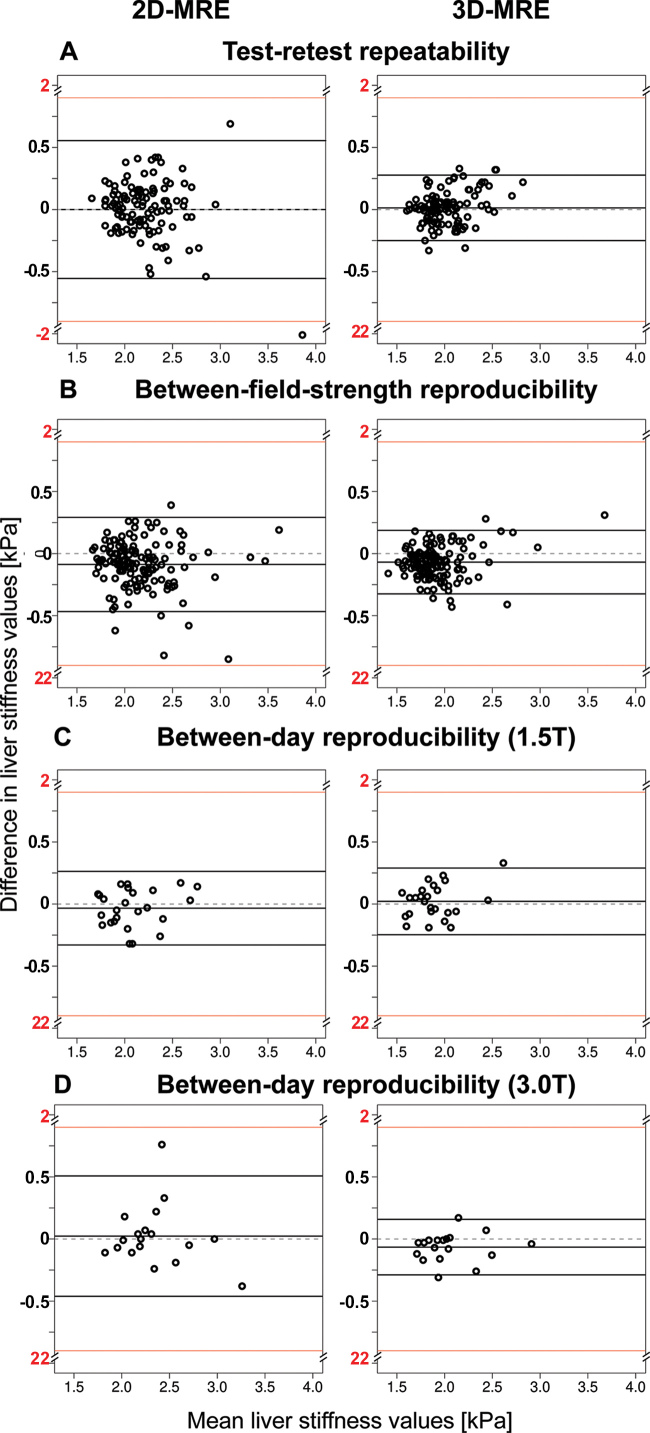
Bland-Altman plots show **(A)** test-retest repeatability, **(B)** between–field-strength reproducibility (1.5 T vs 3 T), and between-day reproducibility at **(C)** 1.5 T and **(D)** 3 T for two-dimensional (2D) MR elastography (MRE) (left) and three-dimensional (3D) MRE liver stiffness (right). The x-axis shows the mean liver stiffness values (in kilopascals) of each measurement pair. The y-axis shows the difference between paired measurements, calculated as follows: **(A)** Second-first measurement, **(B)** 3–1.5 T measurement, and **(C, D)** day 2 to day 1 measurements. Solid horizontal lines indicate the mean difference and the 95% limits of agreement; dashed lines mark zero difference. The interrupted y-axis (break at ±2 kPa, shown in red) compresses the scale to highlight the agreement region while still displaying outliers. Overall, 3D MRE showed better repeatability and reproducibility compared with 2D MRE, with lower repeatability coefficient (RDC) (0.26 kPa vs 0.55 kPa; *P* < .001) and proportional reproducibility coefficient (RC%) (12.6% vs 19.9%; *P* < .001) for test-retest repeatability, lower RDC (0.26 kPa vs 0.38 kPa; *P* < .001) and RDC% (14.6% vs 18.5%; *P* < .001) for between–field-strength reproducibility, and lower repeatability coefficient (RC) for between-day reproducibility at 3 T (0.22 kPa vs 0.48 kPa; *P* = .06).

**Figure 3:**
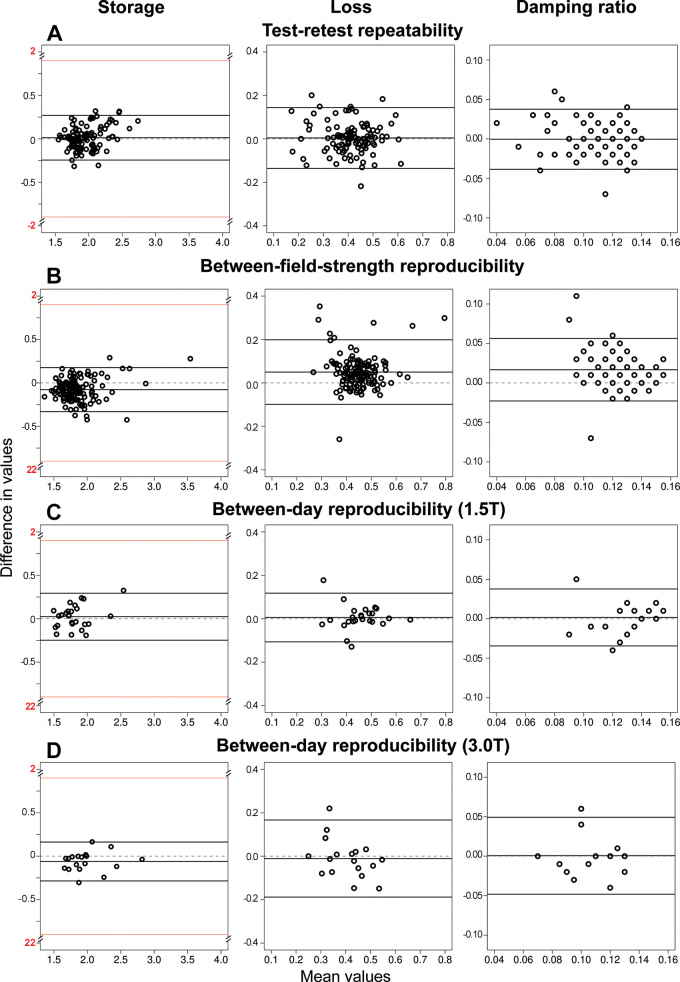
Bland-Altman plots show **(A)** test–retest repeatability, **(B)** between–field-strength reproducibility (1.5 T vs 3 T), and between-day reproducibility at **(C)** 1.5 T and **(D)** 3 T for three-dimensional (3D) MR elastography (MRE) liver stiffness viscoelastic parameters: storage (left), loss (middle), and damping ratio (right). The x-axis shows the mean values of each measurement pair (in kilopascals for storage and loss, damping as a unitless ratio). The y-axis shows the difference between paired measurements, calculated as follows: **(A)** Second-first measurement, **(B)** 3 T to 1.5 T measurement, and **(C, D)** day 2 to day 1 measurements. Solid horizontal lines indicate the mean difference and the 95% limits of agreement; dashed lines mark zero difference. The interrupted y-axis of the storage plots (break at ±2 kPa, shown in red) compresses the scale to highlight the agreement region while still displaying outliers. Overall, 3D MRE storage modulus showed excellent repeatability (repeatability coefficient [RC], 0.26 kPa; proportional RC [RC%], 12.7%; ICC, 0.85), between-field-strength reproducibility (reproducibility coefficient [RDC], 0.25 kPa; proportional RDC [RDC%], 15.5%; intraclass correlation coefficient [ICC], 0.86) and between-day reproducibility for the storage modulus (RDC, 0.27 kPa; RDC%, 14.1%; ICC, 0.85), whereas the loss modulus and damping ratio exhibited larger relative variation at 1.5 T and 3 T.

Test-retest repeatability of MRE at 3 T was assessed in 51 group A and A+ participants (total 110 2D MRE and 107 3D MRE valid datasets) (Table 3; Figs 2, 3).

**Table 3:** Test-Retest Repeatability Measures of 2D and 3D MRE and Viscoelastic Parameters of 3D MRE at 3 T

Biomarker by MRE Method	Mean Bias (kPa)	LOA (kPa)	RC (kPa)	RC% (%)	ICC
Liver stiffness					
2D MRE	0.00	−0.54 to 0.54	0.55 (0.38, 0.95)	19.9 (16.5, 28.0)	0.67 (0.54, 0.78)
3D MRE	0.01	−0.25 to 0.28	0.26 (0.23, 0.30)	12.6 (11.2, 14.2)	0.85 (0.80, 0.90)
*P* Value			<.001	<.001	<.001
Viscoelastic parameters (3D MRE)					
Storage modulus	0.01	−0.24 to 0.27	0.26 (0.22, 0.29)	12.7 (11.2, 14.4)	0.85 (0.79, 0.90)
Loss modulus	0.00	−0.07 to 0.14	0.14 (0.12, 0.16)	41.0 (33.8, 51.8)	0.72 (0.65, 0.80)
Damping ratio	0.00	−0.04 to 0.04	0.04 (0.03, 0.05)	39.7 (32.2, 48.0)	0.60 (0.48, 0.70)

Note.—Data in parentheses are 95% CIs. *P* values indicate statistical significance for pairwise comparisons between two-dimensional (2D) MR elastography (MRE) and three-dimensional (3D) MRE (calculated using bootstrap-based tests). The table shows that, at 3.0 T, 3D MRE provided better test-retest repeatability for liver stiffness than did 2D MRE, with lower repeatability coefficient (RC) and RC% and higher intraclass correlation coefficient (ICC). LOA = limits of agreement, NA = not applicable.

After pooling of data from all visits, mean 2D MRE LS was 2.2 kPa (range, 1.6–4.9 kPa). Mean 3D MRE LS was 2.0 kPa (range, 1.6–2.9 kPa). Compared with 2D MRE, 3D MRE had better repeatability for LS (RC, 0.26 kPa vs 0.55 kPa [*P* < .001]; RC%, 12.6% vs 19.9% [*P* < .001]; ICC: 0.85 vs 0.67 [*P* < .001]).

Mean 3D MRE storage modulus was 2.0 kPa (range, 1.5–2.8 kPa). Test–retest bias was 0.01 kPa (*P* = .29) with limits of agreement of −0.24 to 0.27 kPa, RC of 0.26 kPa, RC% of 12.7%, and ICC of 0.85 (Fig 3). Mean 3D MRE loss modulus was 0.4 kPa (range, 0.1–0.7 kPa). Test–retest bias was 0.00 kPa (*P* = .57) with limits of agreement of −0.13 to 0.14 kPa, RC of 0.14, RC% of 41.0%, and ICC of 0.72. Mean 3D MRE damping ratio was 0.1 (range, 0.03–0.15). Test–retest bias was 0.00 (*P* = .74) with limits of agreement of −0.04 to 0.04, RC of 0.04, RC% of 39.7%, and ICC of 0.60.

### Between–Field-Strength Reproducibility

Between–field-strength reproducibility of MRE was assessed in 53 group B and group B+ participants, and three group A participants who underwent MRE at 1.5 T and 3 T at visit 3, (total 140 2D MRE and 136 3D MRE valid datasets) (Table 4; Figs 2, 3).

**Table 4:** Between–Field-Strength Reproducibility Measures of 2D and 3D MRE and Viscoelastic Parameters of 3D MRE at 3 T

Biomarker MRE Method	Mean Bias	LOA	RDC	RDC% (%)	ICC
Liver stiffness (kPa)					
2D MRE	−0.09	−0.47 to 0.29	0.38 (0.32, 0.46)	18.5 (15.9, 21.7)	0.82 (0.71, 0.91)
3D MRE	−0.07	−0.32 to 0.19	0.26 (0.22, 0.30)	14.6 (13.2, 16.5)	0.88 (0.80, 0.94)
*P* Value	…	…	<.001	<.001	.06
Viscoelastic parameters of 3D MRE					
Storage modulus (kPa)	−0.08	−0.33 to 0.18	0.25 (0.22, 0.30)	15.5 (14.0, 17.5)	0.86 (0.77, 0.94)
Loss modulus (kPa)	0.05	−0.10 to 0.20	0.15 (0.12, 0.19)	45.3 (36.6, 61.2)	0.47 (0.33, 0.60)
Damping ratio	0.02	−0.02 to 0.06	0.04 (0.03, 0.05)	46.4 (37.0, 59.0)	0.19 (0.1, 31.1)

Note.—All metrics are in units of the biomarker except proportional reproducibility coefficient (RDC%). Data in parentheses are 95% CIs. *P* values are for pairwise comparisons between two-dimensional (2D) MR elastography (MRE) and three-dimensional (3D) MRE (calculated using bootstrap-based tests). The table shows that 3D MRE had better between–field-strength reproducibility for liver stiffness than did 2D MRE, with lower absolute reproducibility coefficient (RDC) and RDC% and higher intraclass correlation coefficient (ICC). LOA = limits of agreement, NA = not applicable.

With pooling of data from all visits, mean 2D MRE LS was 2.1 kPa at 1.5 T (range, 1.6–3.7 kPa) and 2.2 kPa at 3 T (range, 1.7–3.5 kPa). Mean 3D MRE LS was 1.9 kPa at 1.5 T (range, 1.3–3.8 kPa) and 2.0 kPa at 3 T (range, 1.5–3.5 kPa). Compared with 2D MRE, 3D MRE had better between–field-strength reproducibility (3D MRE vs 2D MRE: RDC, 0.26 kPa vs 0.38 kPa [*P* < .001]; RDC%, 14.6% vs 18.5% [*P* < .001]; ICC, 0.88 vs 0.82 [*P* = .06]) (Fig 2).

Mean 3D MRE storage modulus was 1.8 kPa at 1.5 T (range, 1.3–3.7 kPa) and 1.9 kPa at 3 T (range, 1.4–3.4 kPa). Between–field-strength bias was −0.08 kPa (*P* < .001) with an RDC of 0.25 kPa, RDC% of 15.5%, and ICC of 0.86 (Fig 3). Mean 3D MRE loss modulus was 0.5 kPa at 1.5 T (range, 0.2–0.9 kPa) and 0.4 kPa at 3 T (range, 0.1–0.7 kPa). Between–field-strength bias was 0.05 kPa (*P* < .001) with an RDC of 0.15 kPa, RDC% of 45.3%, and ICC of 0.47. Mean 3D MRE damping ratio was 0.14 at 1.5 T (range, 0.07–0.17) and 0.12 at 3 T (range, 0.04–0.15). Between–field-strength bias was 0.02 (*P* < .001) with an RDC of 0.04, RDC% of 46.4%, and ICC of 0.19.

### Between-Day Reproducibility

**Figure 4:**
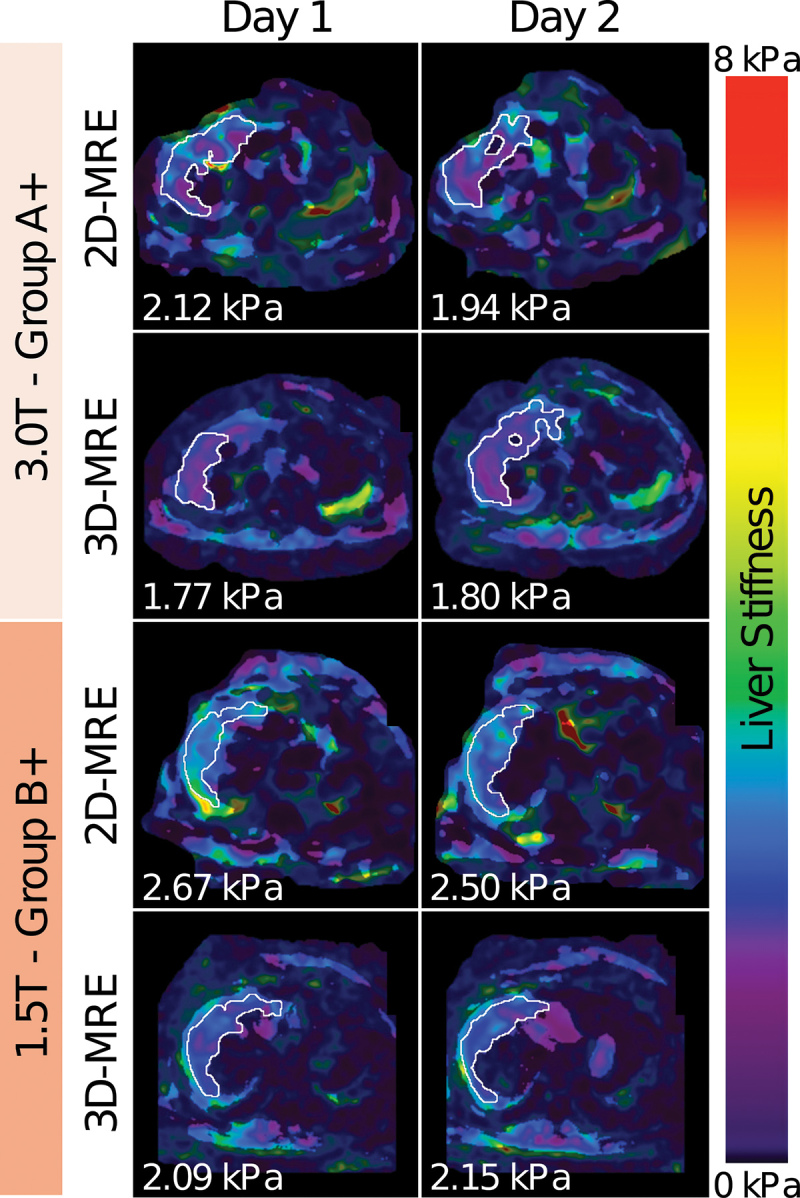
Two-dimensional (2D) MR elastography (MRE) and three-dimensional (3D) MRE sections on the axial plane obtained at 3 T in a 37-year-old woman with obesity assigned to group A+ (top) and at 1.5 T in a 35-year-old man with obesity assigned to group B+ (bottom) show close agreement between liver stiffness measurements performed on different days (1–3 days apart). Automated analysis software measured liver stiffness for each method (2D MRE, 3D MRE) at each time point. The white outline represents the region of interest generated by the software from which liver stiffness values for each pixel were recorded. The mean liver stiffness value is shown on the lower left corner. Liver stiffness values are depicted by color, as shown on the scale bar on the right: Dark purple represents 0 kPa, and red represents 8 kPa (range, 0–8 kPa).

Between-day reproducibility of MRE at 3 T was assessed in 26 group A+ participants (total 18 2D MRE and 18 3D MRE valid datasets) (Table S3; Figs 2–4).

Compared with 2D MRE, 3D MRE had a lower RDC (0.22 kPa vs 0.48 kPa; *P* = .007); there was no evidence of a difference between the two in RDC% (12.4% vs 19.2%; *P* = .19) or ICC (0.92 vs 0.80; *P* = .22).

Between-day reproducibility of MRE at 1.5 T was assessed in 25 group B+ participants (total 25 2D MRE and 25 3D MRE valid datasets). There was no evidence of a difference between 2D MRE and 3D MRE for any precision parameter (all *P* > .05) (Table S3).

### Reproducibility between 2D and 3D MRE

Reproducibility between 2D MRE and 3D MRE for measuring LS was assessed in 51 participants at 3 T (total of 134 2D MRE and 3D MRE valid datasets) and 56 participants at 1.5 T (172 2D MRE and 3D MRE valid datasets) (Fig S3). LS at 2D MRE was 0.22 kPa higher than LS at 3D MRE at each field strength (*P* < .001 for both). Intermethod ICC was 0.56–0.69 and intermethod RDC% was 26.1%–27.4%, depending on field strength.

## Discussion

This prospective dual-center study evaluated the repeatability (at 3 T), between–field-strength reproducibility (at 1.5 T or 3 T), and between-day reproducibility (at 3 T and 1.5 T) of two-dimensional (2D) and three-dimensional (3D) MR elastography (MRE)–based liver stiffness (LS) in adults with severe obesity. We found that 3D MRE has better repeatability (repeatability coefficient [RC], 0.26 vs 0.55 kPa; proportional RC, 12.6% vs 19.9%; intraclass correlation coefficient [ICC], 0.85 vs 0.67; all *P* < .001), superior between–field-strength reproducibility (reproducibility coefficient [RDC], 0.26 vs 0.38 kPa; proportional RDC, 14.6% vs 18.5%; both *P* < .001), and superior between-day reproducibility at 3.0 T (RDC, 0.22 vs 0.48 kPa; *P* = .007) than 2D MRE for LS measurement, but that 3D MRE failed more frequently (4.9% [29 of 587] vs 2.9% [17 of 583]; *P* = .007), particularly at 3.0 T. 3D MRE also demonstrated good precision for measuring storage modulus but poor to fair precision for measuring loss modulus and damping ratio. The 2D MRE and 3D MRE LS measurements differed, consistent with previous studies demonstrating small systematic differences between the two methods (20,29). This discrepancy has been attributed to overestimation by 2D MRE, which analyzes a simplified two-dimensional wavefield, compared with 3D MRE, which incorporates a more complete three-dimensional wavefield. These systematic differences preclude interchangeable use of 2D and 3D MRE for longitudinal follow-up.

Our findings extend previous work by assessing MRE precision in adults with severe obesity, a group for whom published data remain limited. For 2D MRE, earlier studies in healthy volunteers and patients with viral hepatitis reported excellent repeatability and reproducibility, with ICCs ranging from 0.92 to 0.98 (13–17). In contrast, we observed lower ICCs and higher repeatability coefficients for 2D MRE in participants with severe obesity, suggesting that excess adiposity degrades precision relative to nonobese cohorts. For 3D MRE, smaller studies in healthy volunteers and patients with mixed chronic liver diseases reported ICCs greater than 0.80 (30,31), which is consistent with our results in severe obesity. Previous direct comparative studies of 2D MRE versus 3D MRE in healthy volunteers (32) and patients without obesity (33) did not find superior 3D MRE precision. Our results, however, show a consistent precision advantage of 3D MRE over 2D MRE in severe obesity, across test–retest, between-day, and between–field-strength settings. In direct comparison, 2D MRE and 3D MRE did not significantly differ in diagnostic accuracy for fibrosis staging in a cohort with a maximum body mass index of 34 (measured in kilograms of body weight divided by height in meters squared) (20). The comparative accuracy of 2D and 3D MRE in populations with severe obesity has not been examined and is a future direction. However, even in the absence of cross-sectional accuracy differences, improved precision may reduce measurement variability around clinically relevant LS thresholds, avoiding misclassification of patients.

Regarding viscoelastic parameters, previous reports focused on diagnostic performance (27,34,32); to our knowledge, our study is among the first to assess precision in severe obesity. We found that storage modulus exhibited precision metrics similar to LS, whereas loss modulus and damping ratio showed substantially lower ICCs, consistent with earlier reports in mixed populations that suggested limited robustness of these parameters (24,35).

In our study sample, the technical failure rate of 2D MRE was slightly lower than in previous reports of 3%–15% in populations without obesity at 1.5 and 3 T (33,36,37). The failure rate of 3D MRE was slightly higher than that of 2D MRE, whereas previous studies generally reported similar or lower failure rates for 3D MRE (31,36). This discrepancy may reflect the specific challenges of severe obesity at 3 T, including lower signal-to-noise ratio and attenuated wave transmission through the abdominal wall, underscoring the need for technical refinements to reduce 3D MRE failure rates in this setting.

Taken together, our findings indicate that 3D MRE provides superior precision for LS compared with 2D MRE, but with the trade-off of slightly higher technical failure rate and the need for more breath-holds. On the basis of the totality of current evidence, we do not recommend discarding 2D MRE; rather, 3D MRE could be selected in settings where maximal precision is critical (eg, multicenter clinical trials or longitudinal monitoring of therapeutic response), whereas 2D MRE may be an adequate and practical alternative when 3D MRE is unavailable or contraindicated.

Users of 3D MRE should also be aware that 3D MRE–based LS is slightly lower than 2D MRE–based LS, which will necessitate the identification and validation of new classification thresholds. The superior precision of 3D MRE is unlikely to be explained by its larger volumetric coverage because our analysis was restricted to four central sections; instead, it likely reflects the full three-directional encoding of wave motion, a hypothesis that warrants further mechanistic investigation.

Conversely, the higher failure rate of 3D MRE may result from the requirement for multiple breath-holds, thinner sections, greater motion sensitivity, or reconstruction challenges, and future technical refinements should address these limitations. Breath-hold burden is another barrier to widespread adoption, but free-breathing 3D MRE sequences under development could mitigate this issue and broaden its clinical applicability. With respect to viscoelastic parameters, the storage modulus shows sufficient precision to be considered in research and possibly clinical contexts, although the incremental value of measuring the storage modulus in addition to LS is not yet clear. The loss modulus and damping ratio require technical improvement and multicenter standardization before they can serve as reliable endpoints in clinical trials.

Overall, our study underscores the potential of 3D MRE to enhance quantitative liver imaging in severe obesity, while also delineating the challenges that must be overcome before integration into routine clinical care. Strengths of our study were its prospective, dual-center design, and the use of automated quantitative analysis to minimize operator dependency.

Our study had limitations. First, although it reflects real-world demographic characteristics of individuals seeking care in bariatric clinics, it may not represent the broader population with MASLD or MASH. In particular, our study sample was predominantly female and had a relatively low prevalence of significant fibrosis and, therefore, of high LS values. Thus, the generalizability of our findings to men or to populations with more advanced liver disease may be limited. Second, the number of participants contributing data for between-day reproducibility analysis of MRE at 3.0 T was small, which may limit interpretation of those specific data. Third, we used MRI systems from a single vendor, although with multiple MRI system models and at two sites. In addition, MRE examinations were performed by multiple technologists, which may introduce operator-related variability despite standardized training and protocols. However, this reflects real-world clinical practice, where MRE examinations are likely to be performed by different operators. Finally, MRE data were analyzed with an automated pipeline. Although this represents the cutting edge and likely the future of MRE, our findings may not directly translate to settings where manual analysis is used, which is more time-consuming, prone to operator variability and potentially less reproducible.

In conclusion, three-dimensional (3D) MR elastography (MRE) demonstrated better test-retest repeatability, between–field-strength reproducibility, and between-day reproducibility at 3 T when measuring liver stiffness in adults with severe obesity, but it failed more frequently. These results support the potential role of 3D MRE as a robust quantitative imaging method for liver tissue characterization in obesity. However, its slightly higher technical failure rate and greater breath-hold burden with more and longer breath-holds during image acquisition remain barriers that must be addressed before widespread clinical adoption. For now, 2D MRE remains an acceptable and practical alternative when 3D MRE is not available or feasible.

## Supplemental Files

Appendix S1, Tables S1-S3, Figures S1-S3

Conflicts of Interest
