# Supplementary material for: Comparative Precision of 3D MRE and 2D MRE for Measurement of Liver Stiffness in Adults with Severe Obesity
Source: Radiology. 2026 May 5;319(2):e253243. doi: 10.1148/radiol.253243 (PMC13216705; doi:10.1148/radiol.253243)
Supplement: Appendix S1, Tables S1-S3, Figures S1-S3 [file ry253243supp.pdf]

Supplemental materials undergo peer review but are not copyedited, and may include typographical errors.

©RSNA, 2026
10.1148/radiol.253243

Appendix S1

Supplemental Materials

Bariatric surgery and histopathology

Experienced surgeons (A.L., finished residency in 2003; L.F., finished residency in 2012; G.J., finished residency in 2007) performed bariatric surgery and, for research purposes, acquired an intraoperative wedge biopsy of the left liver lobe. Two expert hepatopathologists (R.A., finished residency in 2001, and R.S., finished residency in 2022 and joined the study at the end for the final consensus reads) scored the histology slides in consensus (Non-alcoholic steatohepatitis Clinical Research Network system) (20) blinded to other study data.

Clinical and laboratory assessment

Demographic data and blood tests were collected at Visit 1. Weight and height were measured at each visit. Body mass index was calculated.

Table S1: Overview of the acquisition sequences and parameters used for MRE

Parameter	2D-MRE	3D-MRE
Pulse sequence	SE EPI	SE EPI
Plane	Axial	Axial
Position	Supine	Supine
Image acquisition mode	2D	2D
TR (ms)	1600	1600
TE (ms)	Min. Full (~55)	Min. Full (~55)
Number of breath-holds(s)	1	6
Acquisition time (min)	0:16	1:17
Flip angle	90°/180°	90°/180°
Field of view (cm)	44x44	44.8x44.8
Matrix	96x96	96x96
Readout direction	Right-Left	Right-Left
Slice thickness (mm)	8	3.5
Inter-slice gap (mm)	2	0
Number of slices	4	4

Supplemental materials undergo peer review but are not copyedited, and may include typographical errors.

Motion-sensitization direction	z	x,y,z
MEG frequency (Hz), Amplitude (µm)	60, 70	60, 70
MEG amplitude scale factor	1	1
Motion Encoding Directions	SI	RL / AP / SI

AP, anterior-posterior; MEG, motion encoding gradients; RL, right-left; SI, superior-inferior; TR, repetition time; TE, echo time.

Supplemental materials undergo peer review but are not copyedited, and may include typographical errors.

Table S2. Weight and BMI values over visits

Visit	Weight (kg)	BMI (kg/m^2)
1+	134.9 ± 25.9	47.4 ± 7.6
1	129.3 ± 25.1	45.9 ± 7.4
2	124.9 ± 24.1	44.6 ± 7.2
3	98.4 ± 22.4	34.9 ± 6.5

BMI, body mass index. Shown are mean ± standard deviation.

Supplemental materials undergo peer review but are not copyedited, and may include typographical errors.

Table S3. Between-day reproducibility measures of 2D- and 3D-MRE and the viscoelastic parameters of 3D-MRE at 3.0T and 1.5T

**Biomarker (unit)**	**Field strength**	**MRE Method**	**Mean bias** (Day 2-Day 1) (kPa)	**LOA** (Day 2-Day 1) (kPa)	**RDC** (kPa)	**P-value**	**RDC%**	**P-value**	**ICC**	**P-value**
Liver Stiffness (kPa)	3.0T	2D-MRE	0.02	-0.46-0.51	0.48 (0.28-0.76)	.007	19.2 (11.7-34.5)	.19	0.80 (0.33-0.93)	.22
		3D-MRE	-0.07	-0.29-0.16	0.22 (0.16-0.32)		12.4 (8.8-18.2)		0.91 (0.75-0.98)	
	1.5T	2D-MRE	-0.03	-0.32-0.26	0.30 (0.24-0.37)	.53	14.3 (11.5-18.2)	.71	0.87 (0.73-0.95)	.85
		3D-MRE	0.02	-0.25-0.29	0.26 (0.21-0.35)		13.5 (10.8-16.1)		0.86 (0.72-0.93)	
Viscoelastic parameters										
Storage modulus (kPa)	3.0T	3D-MRE	-0.06	-0.28-0.16	0.22 (0.16-0.31)	—	12.7 (9.1-18.2)	—	0.92 (0.75-0.98)	—
Loss modulus (kPa)			-0.01	-0.19-0.17	0.18 (0.12-0.28)	—	46.6 (31.2-73.0)	—	0.58 (0.19-0.82)	—
Damping ratio			0.00	-0.05-0.05	0.05 (0.03-0.07)	—	46.2 (31.2-67.0)	—	0.29 (-0.18-0.73)	—
Storage modulus (kPa)	1.5T	3D-MRE	0.02	-0.25-0.29	0.27 (0.22-0.35)	—	14.1 (11.5-16.9)	—	0.85 (0.70-0.93)	—
Loss modulus (kPa)			0.00	-0.11-0.12	0.11 (0.07-0.17)	—	30.5 (17.5-53.3)	—	0.78 (0.54-0.94)	—
Damping ratio			0.00	-0.03-0.04	0.04 (0.03-0.05)	—	31.6 (21.4-52.9)	—	0.56 (0.28-0.77)	—

LOA = limits of agreement. RDC = absolute reproducibility coefficient. RDC% = proportional reproducibility coefficient. ICC = intraclass correlation coefficient. All metrics are in units of the biomarker except RDC% (expressed as a percentage). Shown are p-values for indicated pairwise comparisons between 2D-MRE and 3D-MRE.

Supplemental materials undergo peer review but are not copyedited, and may include typographical errors.

Figure S1. Example of 2D-MRE acquired in the axial plane at 1.5T in a 41-year-old female with obesity, where the first acquisition was a technical failure (first row). The automated analysis software identified zero analyzable pixels. In this case, the technologist did not connect the tubing between the active and passive driver, and so no vibrations were transmitted into the body. Notice flat appearance of the phase images (2nd column), absence of planar wave motion on the wave image (3rd column), and absence of any analyzable pixels over the region of the liver in the elastogram (analyzable pixels are shown in bright colors; non-analyzable pixels are shown in muted colors) (4th column). The exam was repeated successfully (second row). The white outline depicts the region of interest from which liver stiffness measurements were extracted. 2D: two-dimensional, MRE: magnetic resonance elastography.

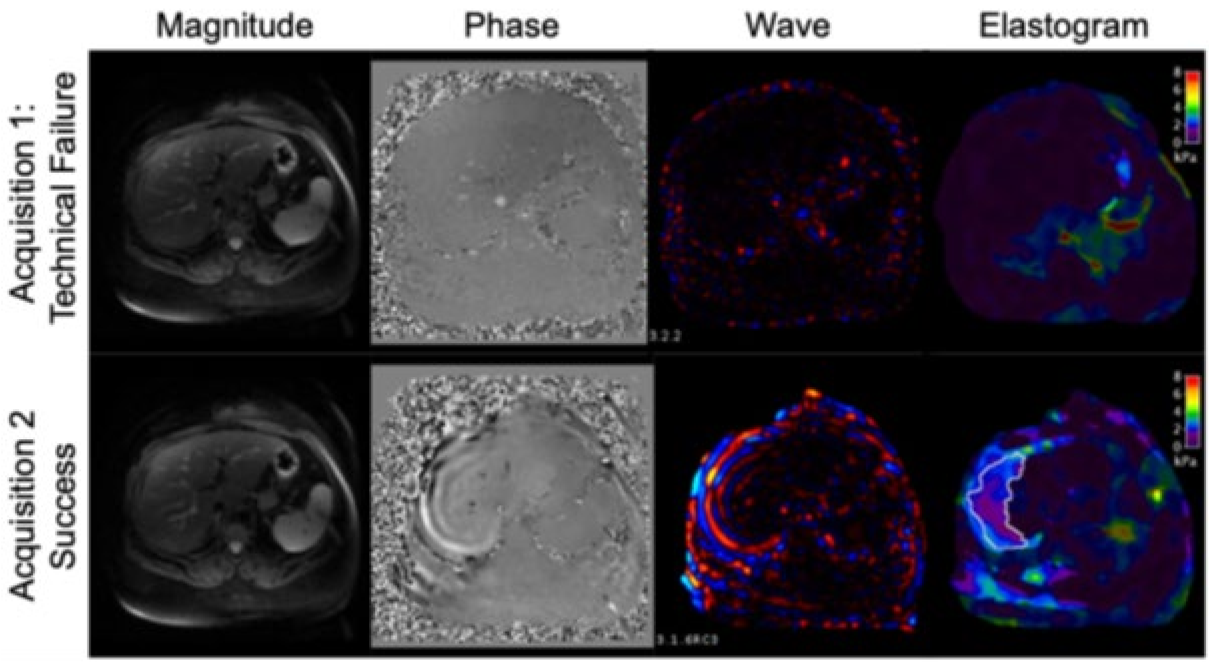

Supplemental materials undergo peer review but are not copyedited, and may include typographical errors.

Figure S2. Failed 2D-MRE (elastography slices in the axial plane acquired at 3.0 T) in a 60-year-old male (top row) and failed 3D-MRE (elastography slices in the axial plane acquired at 3.0 T) in a 38-year-old male (bottom row). The phase (second column) and wave (third column) images show incoherent rather than planar wave propagation, and the elastograms (fourth column) contain fewer than 500 analyzable pixels (analyzable pixels are shown in bright colors; non-analyzable pixels are shown in muted colors). Both acquisitions were classified as technical failures and excluded from analysis, with no LS values reported. 2D: two-dimensional; 3D: three-dimensional; MRE: magnetic resonance elastography; LS: liver stiffness.

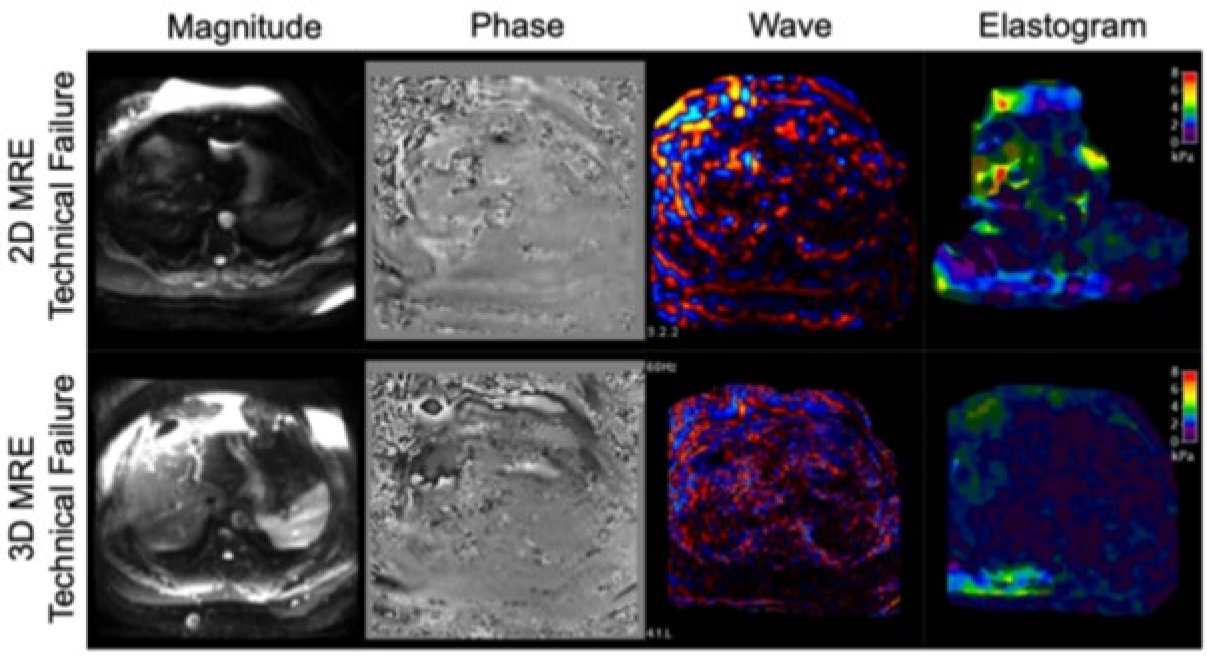

Supplemental materials undergo peer review but are not copyedited, and may include typographical errors.

Figure S3. Bland–Altman plots illustrating agreement between 2D- and 3D-MRE liver stiffness measurements at at 1.5T (A) and 3.0T (B).

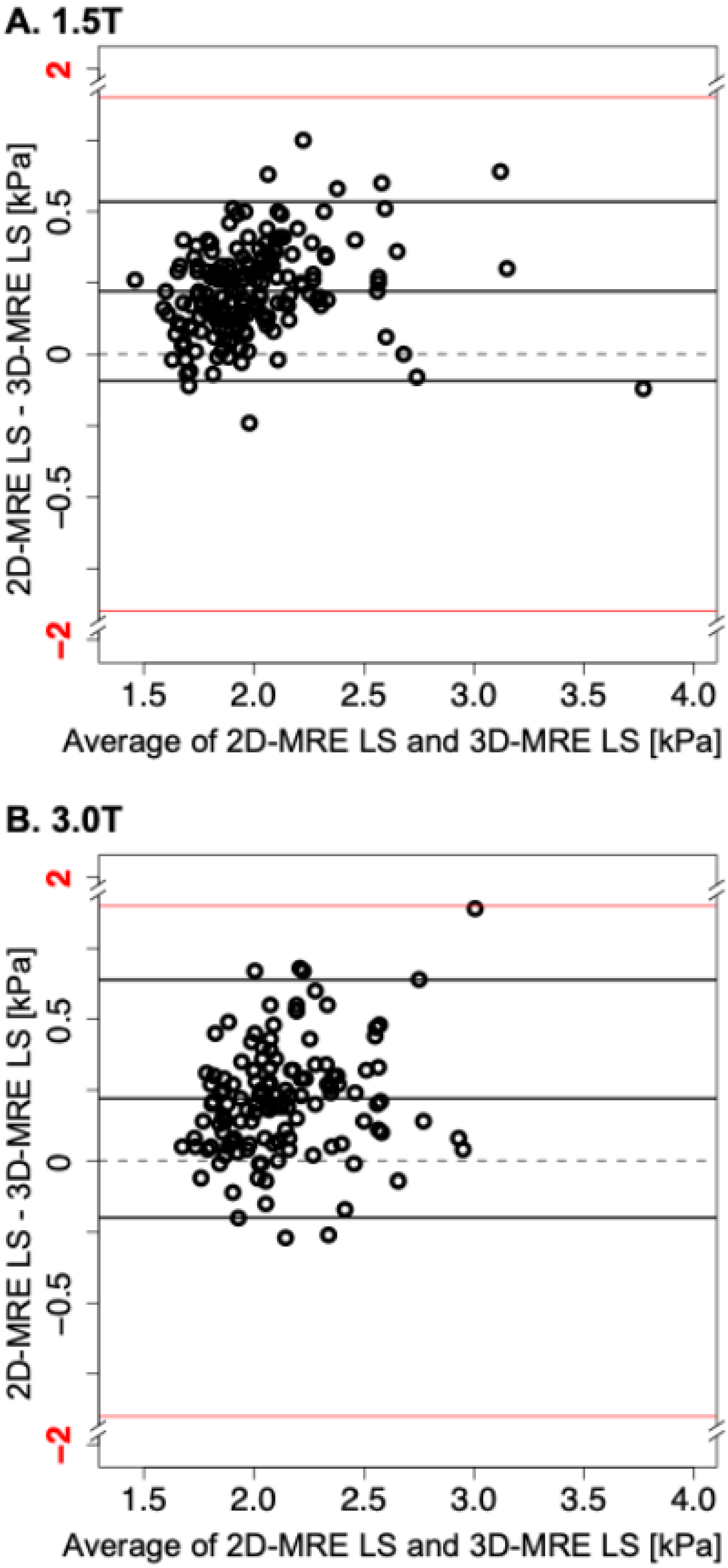

Supplemental materials undergo peer review but are not copyedited, and may include typographical errors.

Bland–Altman analysis comparing LS measured by 2D-MRE and 3D-MRE at 1.5T (A) and 3.0T (B) (2D - 3D measurements) in 56 participants with a total of 172 valid 2D- and 3D-MRE datasets at 1.5T and in 51 participants with a total of 134 valid 2D- and 3D-MRE datasets at 3.0T. Solid horizontal lines indicate the mean difference and the 95% limits of agreement; dashed lines mark zero difference. The interrupted y-axis of the plots (break at ±2 kPa) are provided to maintain consistency with the plots in the main body of the manuscript. There was a small systematic difference in 2D-MRE and 3D-MRE liver stiffness measurements at 1.5T and 3.0T. 2D-MRE values were higher than 3D-MRE values by 0.22 kPa at 1.5T and 3.0T; both $P<.001$*)*. Inter-method ICC was 0.69 at 1.5T and 0.56 at 3.0T and inter-method RDC% was 26.1% at 1.5T and 27.4% at 3.0T.

MRE = magnetic resonance elastography; LS = liver stiffness; ICC = intraclass correlation coefficient; LOA = limits of agreement; SD = standard deviation; RDC% = relative reproducibility coefficient, RDC = absolute reproducibility coefficient; CV = coefficient of variation.
