# Supplementary material for: Comparative Precision of 3D MRE and 2D MRE for Measurement of Liver Stiffness in Adults with Severe Obesity
Source: Radiology. 2026 May 5;319(2):e253243. doi: 10.1148/radiol.253243 (PMC13216705; doi:10.1148/radiol.253243)
Supplement: Conflicts of Interest [file radiol253243coi.zip › 566487858_617599_1770731250264.pdf]

ICMJE DISCLOSURE FORM

Instructions

In the interest of transparency, we ask you to disclose all employment/relationships/activities/interests listed below that are related to the content of your manuscript. “Related” means any relationship with for-profit or not-for-profit third parties whose interests may be affected by the content of the manuscript. Disclosure represents a commitment to transparency and does not necessarily indicate a bias. If you are in doubt about whether to list an employment/relationship/activity/interest, it is preferable that you do so.

The following questions apply to your employment/relationships/activities/interests as they relate to the **current manuscript only**. Each author is required to submit a separate form and is responsible for the accuracy and completeness of the submitted information.

Your employment/relationships/activities/interests should be **defined broadly**. For example, if your manuscript pertains to the epidemiology of hypertension, you should declare all relationships with manufacturers of antihypertensive medication, even if that medication is not mentioned in the manuscript.

Date: 10-Feb-2026

req First Name: Claude

req Last Name: Sirlin

Manuscript Title: Comparative precision of 3D-MRE and 2D-MRE for measurement of liver stiffness in adults with severe obesity

Manuscript number: RAD-25-3243.R1

In item #1 below, report all support for the work reported in this manuscript without time limit. For all other items, the time frame for disclosure is the past 36 months. Note: All items #1 through #13 must indicate none (by checking the box next to None) or include relevant disclosure information in the text boxes. Blank rows will cause the form to be sent back for completion.

	**Name all entities with whom you have this relationship or check the box next to None**	**Specifications/Comments (e.g., if payments were made to you or to your institution)**
**Time frame: Since the initial planning of the work**		
1. All support for the present manuscript (e.g., funding, provision of study materials, medical writing,	☐	None

article processing charges, etc.)<br>**No time limit for this item.**		
	Research funding<br>NIH<br>Pfizer<br><br>In-kind analysis<br>Resoundant<br><br>General research support, not specific to the paper<br>GE	All payments and support to institution
**Time frame: past 36 months**		
2. Grants or contracts from any entity (if not indicated in item #1 above).	☐	None
	Research grants<br>ACR<br>Bayer<br>GE<br>Pfizer<br>Gilead<br>Philips<br>Siemens<br>V Foundation<br><br>Lab service agreements<br>OrsoBio<br>Enanta<br>Gilead<br>ICON<br>Intercept<br>Nusirt<br>Prosciento<br>Shire<br>Synageva<br>Takeda<br>Velocity	payments to institution
3. Royalties or licenses	☐	None
	Medscape<br>Wolters Kluwer	Payments to self
4. Consulting fees	☐	None
	Personal consulting<br>Boehringer	Personal consulting<br>Payments to self

	Ingelheim<br>Altimmune<br>Ascelia Pharma AB<br>Blade Epigenomics<br>Guerbet<br>Livivos<br><br>Institutional consulting<br>AMRA<br>Bayer<br>Exact Sciences<br>Pfizer	Institutional consulting payments to institution
5. Payment or honoraria for lectures, presentations, speakers bureaus, manuscript writing or educational events	☐	None
	Educational symposia<br>University of Cincinnati<br>University of Mississippi<br>Sociedad Radiológica de Puerto Rico	Payments to self
6. Payment for expert testimony	✓	None
7. Support for attending meetings and/or travel	☐	None
	Fundacion Santa Fe<br>Congreso Argentino de Diagnóstico por Imágenes<br>Stanford<br>Jornada Paulista de Radiologia<br>Ascelia Pharma AB<br>University of Cincinnati<br>University of Mississippi<br>RSNA<br>Sociedad Radiológica de Puerto Rico	Reimbursement for travel to me

	Hospital Español Auxilio Mutuo, Puerto Rico	
8. Patents planned, issued or pending	✓	None
9. Participation on a Data Safety Monitoring Board or Advisory Board	☐	None
	NCI- National Cancer Institute funded Early Detection Research Network	Unpaid
10. Leadership or fiduciary role in other board, society, committee or advocacy group, paid or unpaid	☐	None
	Livivos executive position for Livivos (Chief Medical Officer, unsalaried position with stock options and stock) through June 28, 2023; Principal Scientific Advisor to Livivos (unsalaried position with stock options and stock) since June 28, 2023	Stock and tock options to me
11. Stock or stock options	☐	None
	Livivos	Stock and tock options to me
12. Receipt of equipment, materials, drugs, medical writing, gifts or other services	☐	None
	Equipment loan (GE LOGIQ E10) Equipment	Equipment, material, and services to university

	Loan (Siemens Sequoia) Equipment Loan (MRE technology from GE) Equipment Loan (MRE technology from Mayo) Equipment Loan (Butterfly IQ probes) In-kind research support (research analysis by Perspectum) In-kind research support (research analysis by AMRA) Provision of drug (contrast material [Eovist] from Bayer)	
13. Other financial or non-financial interests	✓	None

req **Please check the box next to the following statement to indicate your agreement:**

✓ I certify that I have answered every question and all the information is complete and accurate.

This is a reprint of the ICMJE Recommendations for the Conduct, Reporting, Editing and Publication of Scholarly Work in Medical Journals. RSNA prepared this reprint. The ICMJE has not endorsed nor approved the contents of this reprint. The official version of the Recommendations for the Conduct, Reporting, Editing and Publication of Scholarly Work in Medical Journals is located at www.ICMJE.org. Users should cite this official version when citing the document.
